# The Royal British Nurses' Association

**Published:** 1917-10-20

**Authors:** Constance Emily Campbell Thomson, Herbert J. Paterson, Isabel Macdonald

**Affiliations:** Nurse Honorary Secretary. 10 Orchard Street, W.; Medical Honorary Secretary. 10 Orchard Street, W.; Secretary. 10 Orchard Street, W.


*To the Editor of* The Hospital.

### The Royal British Nurses' Association.

*To the Editor of* The Hospital.

Sir,—Inquiries have reached us from certain members of the Royal British Nurses' Association in Nottingham and elsewhere as to a statement which appeared in the recent press reports of a meeting held under the auspices of the College of Nursing, Limited, in that town. The Secretary of the College of Nursing, Limited, is reported to have stated at this meeting that "more than a year ago another Society, the Royal British Nurses' Association, approached the College of Nursing with a view to *affiliation.*" We can best deal with this statement by saying at once that it is misleading and inaccurate. The facts are that the College of Nursing, Limited, approached the Royal British Nurses' Association with a view to its *amalgamation* with the latter body. To the member who quoted from one press report of the speech to the effect that the Secretary had "pointed out that the Royal British Nurses' Association wished to affiliate with the College" we would reply, that the statement bears with it its own contradiction. How, we ask, could an Association, having a Royal President, and incorporated by Royal Charter, propose to affiliate itself to a body incorporated under the Companies Act?

The speaker appears to have arrived at the conclusion, again to quote her words as reported, that "the Association might be fearful lest its identity, however well safeguarded, should be lost." When such misleading statements as that quoted come to their notice we think that our members have just cause for anxiety as to whether the prestige of their Association has been sufficiently "well safeguarded." Moreover, such statements must lead to confusion in the minds of our members, for, at the verbal request of the Chairman of the College of Nursing, Limited, it was pointed out to them at their general meeting that the College was to be amalgamated with the Association, and not the Association with the College of Nursing, Limited.—We are, etc.,

(Signed) Constance Emily Campbell Thomson,
Nurse Honorary Secretary.
Herbert J. Paterson,
Medical Honorary Secretary.
Isabel Macdonald,
Secretary.

10 Orchard Street, W.,
October 16, 1917.

*** See Note in "Round the Hospitals," page 57.

